# Preparation and Characterization of a Polyclonal Antibody against Human Actin Filament-Associated Protein-120 kD

**DOI:** 10.3390/ijms17060942

**Published:** 2016-06-17

**Authors:** Yujian Chen, Yong Liu, Jiayu Guo, Tao Tang, Jian Gao, Tao Huang, Bin Wang, Shaojun Liu

**Affiliations:** Department of Neurobiology, Institute of Basic Medical Sciences, Beijing 100850, China; triser@126.com (Y.C.); gjy992@outlook.com (J.G.); taotang006@163.com (T.T.); gaojian1918@163.com (J.G.); huang_tao945@163.com (T.H.); xiechuanbin2003@163.com (B.W.)

**Keywords:** AFAP-120, polyclonal antibody, NINS fragment, AFAP-110, B-cell epitope

## Abstract

Actin filament-associated protein-120kD (AFAP-120) is an alternatively spliced isoform of actin filament-associated protein-110kD (AFAP-110) and contains an additional neuronal insert (NINS) fragment in addition to identical domains to the AFAP-110. Unlike AFAP-110 widely expressed in tissues, AFAP-120 is specifically expressed in the nervous system and plays a role in organizing dynamic actin structures during neuronal differentiation. However, anti-AFAP-120 antibody is still commercially unavailable, and this may hinder the function research for AFAP-120. In this study, we simultaneously used the ABCpred online server and the BepiPred 1.0 server to predict B-cell epitopes in the exclusive NINS sequence of human AFAP-120 protein, and found that a 16aa-peptide sequence was the consensus epitope predicted by both tools. This peptide was chemically synthesized and used as an immunogen to develop polyclonal antibody against AFAP-120 (anti-AFAP-120). The sensitivity and specificity of anti-AFAP-120 were analyzed with immunoblotting, immunoprecipitation, and immunofluorescence assays. Our results indicated that anti-AFAP-120 could react with over-expressed and endogenous human AFAP-120 protein under denatured condition, but not with human AFAP-110 protein. Moreover, native human AFAP-120 protein could also be recognized by the anti-AFAP-120 antibody. These results suggested that the prepared anit-AFAP-120 antibody would be a useful tool for studying the biochemical and biological functions of AFAP-120.

## 1. Introduction

Actin filament-associated protein-110kD (AFAP-110), originally found as a substrate and binding partner of Src kinase and protein kinase C alpha (PKCα), is an actin filament-crosslinked protein [1,2,3,4]. Subsequently, an alternatively spliced isoform of AFAP-110, actin filament-associated protein-120kD (AFAP-120), is identified. AFAP-110 and -120 (AFAPs) are multi-domain proteins containing two pleckstrin homology domains (PH) for interacting with PKCα [5,6,7], SH2, and SH3 motifs for binding cSrc [8,9] and carboxy-terminal leucine zipper (Lzip), which mediate AFAP oligomerization [2,10] and interactions with F-actin. AFAP-120 contains an additional neuronal insert (NINS) near the carboxy terminus which does not disrupt the reading frame of the downstream coding sequence [11].

The staining of mouse brain sections with an antiserum against AFAPs demonstrated that AFAPs are widely expressed in embryonic and early postnatal brain regions including the cortex, forebrain, cerebellum, and olfactory bulb [12]. AFAPs expression levels were dramatically decreased in the adult brain, with a high concentration only detected in the olfactory bulb [12]. With Northern and Western blot (using phospho-AFAP-specific antiserum) analysis, it was shown that AFAP-110 is expressed in most tissues, but AFAP-120 is specifically expressed in the nervous system [2,11]. In neurons, AFAP-120 is enriched in the axon and growth cone, and more abundant and more highly tyrosine phosphorylated than AFAP-110 [2,13].

So far, it is well-known that AFAP-110 plays a critical role in the regulation of the formation and maintenance of actin cytoskeleton [6,14], the function of focal contact [14,15], podosome formation and invasion [10,16,17], and cell migration [18]. However, relatively little is known about the functions of neuronal-specific AFAP-120 in the nervous system, especially about the functions of the NINS fragment. In general, a specific antibody is a powerful tool to study the function of protein. Unfortunately, the AFAP-120-specific antibody is still commercially unavailable. Although the existing anti-AFAP antibody can recognize the AFAP-120 protein, it has several disadvantages when used to study the function of the AFAP-120 protein, such as the undistinguished binding to AFAP-110 and AFAP-120 proteins in immunoprecipitation and immunofluorescence assays.

In this study, we predicted B-cell epitopes in the additional NINS fragment of the human AFAP-120 protein and selected a peptide containing 16 amino acids as an immunogen. The polyclonal antibody against human AFAP-120 was produced by immunizing rabbit with this immunogen. The sensitivity and specificity of anti-AFAP-120 were analyzed with immunoblotting, immunoprecipitation, and immunofluorescence assays. These results suggested that the prepared antibody had an excellent immunoreactivity and would be useful in understanding the function of the AFAP-120 protein.

## 2. Results

### 2.1. Sequence Analysis and AFAP-120 Protein Epitope Prediction

**Figure 1:**
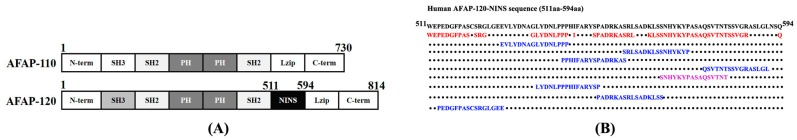
Sequence analysis and prediction of AFAP-120 protein B-cell epitopes. (**A**) Human AFAP-110 (730 aa) and AFAP-120 (814 aa) proteins contain identical SH3 binding domains, two SH2 binding domains, two pleckstrin homology (PH) domains, and a leucine zipper (Lzip) motif. AFAP-120 contains an additional neuronal insert (NINS) (511–594 aa), generated by alternative splicing; (**B**) B-cell epitopes in the NINS sequence were simultaneously predicted by the ABCpred online server and the BepiPred 1.0 server. The black letters represent the sequence of NINS (from 511 to 594 aa). The blue letters represent the epitopes predicted by the ABCpred online server. The red letters represent the epitopes predicted by the BepiPred 1.0 server. The purple letters represent the common epitope predicted by both tools. The black dots represent non-epitopes predicted by both tools.

AFAP-110 and AFAP-120 are multi-domain proteins that contain the same domains except the NINS (511–594 aa) (Figure 1A). To develop an antibody specifically recognizing AFAP-120, we simultaneously predicted the B-cell epitopes of human NINS using the ABCpred online server and the BepiPred 1.0 server. The results showed that eight epitopes (Figure 1B, blue letters) were predicted by the ABCpred online server, and seven epitopes (Figure 1B, red letters) by the BepiPred 1.0 server. In those predicted epitopes, only a 16aa-peptide sequence (SNHYKYPASAQSVTNT) was the consensus epitope predicted by both tools (Figure 1B, purple letters). This 16aa-peptide was selected as immunogen.

### 2.2. Identification and Purification of the Synthetic Epitope Peptide

**Figure 2:**
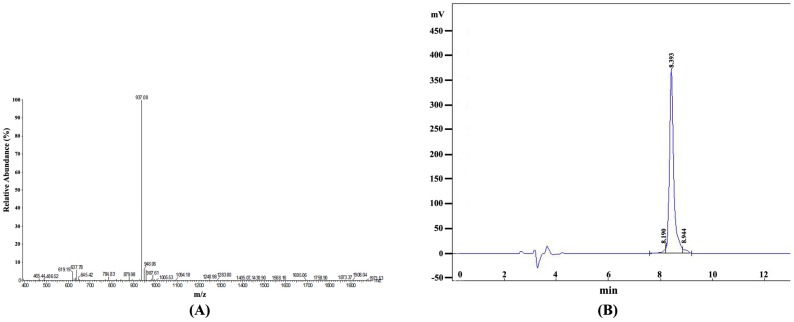
Identification and purification of the synthetic epitope peptide. (**A**) Mass-spectrum graph of the synthetic peptide; (**B**) Purity identification of the synthetic peptide by HPLC.

After being synthesized, the 16aa-peptide was identified by mass spectrum (MS). The result showed that the molecular weight of synthetic peptide was 1871.02 Dalton, and its characteristics of MS accorded well with the theoretical value of this 16aa-peptide sequence (Figure 2A). When the synthetic peptide was purified with high performance liquid chromatography (HPLC) (Figure 2B), the purity was 95.2168% (calculated by peak area) (Figure 2B, Table 1). This synthetic peptide was conjugated with the carrier protein KLH and was then used to immunize a rabbit to produce the anti-AFAP-120 polyclonal antibody.

### 2.3. Recognization of the Anti-AFAP-120 Antibody to Human NINS Peptide

**Figure 3:**
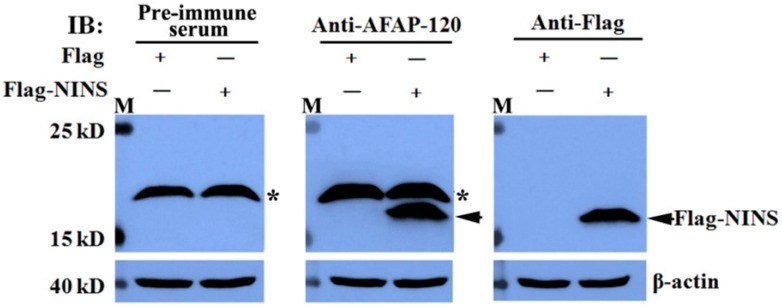
The anti-AFAP-120 antibody recognized human NINS peptide. HEK 293T cells were transfected with empty vector pCMV-Flag (Flag) or pCMV-Flag-human NINS (Flag-NINS) plasmids for 48 h. The extracted cell proteins were immunoblotted with antibodies against AFAP-120 (anti-AFAP-120) or Flag tag (anti-Flag), respectively. Rabbit pre-immune serum was used as a negative control and β-actin as the internal control. Black solid arrows indicate specifically-probed Flag-NINS. Black asterisks indicate nonspecific recognization of antibodies.

To detect whether human NINS could be immunoblotted by the anti-AFAP-120 antibody, plasmids pCMV-Flag-human NINS and empty vector pCMV-Flag were transfected into HEK293T cells for 48 h. The cells were lysed, and the extracted proteins were immunoblotted with antibodies against AFAP-120 and Flag tag, respectively. Meanwhile, the rabbit pre-immune serum was used as a negative control. The results indicated that over-expressed Flag-tagged human NINS could be immunoblotted by anti-AFAP-120 and anti-Flag antibodies (Figure 3), but not by pre-immune serum (Figure 3).

### 2.4. Recognization of the Anti-AFAP-120 Antibody to Human Denatured AFAP-120

**Figure 4:**
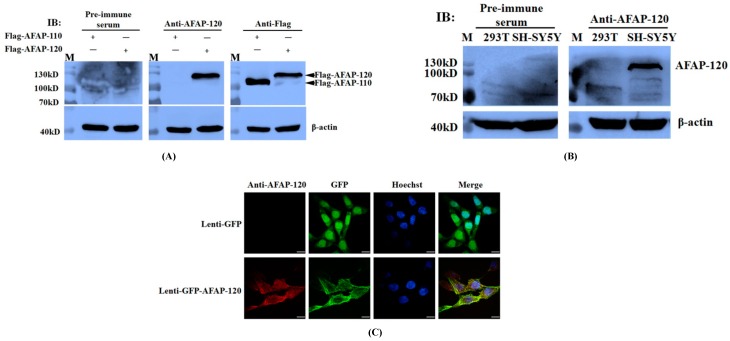
Recognization of anti-AFAP-120 antibody to denatured human AFAP-120. (**A**) HEK 293T cells were transfected with plasmids pCMV-Flag-human AFAP-120 and pCMV-Flag-human AFAP-110 for 48 h. The extracted cell proteins were immunoblotted with antibodies against AFAP-120 (anti-AFAP-120) or Flag tag (anti-Flag) respectively. Rabbit pre-immune serum was used as a negative control and β-actin as the internal control. Black solid arrows indicate the corresponding AFAP protein; (**B**) HEK293T and SH-SY5Y cells were lysed and 25 μg of whole cell protein lysate was subjected to immunoblotting assay with anti-AFAP-120. Pre-immune serum was used as a negative control and β-actin as the internal control; (**C**) COS-7 cells were infected with recombinant lentiviruses expressing GFP (lenti-GFP) or GFP-human AFAP-120 (lenti-GFP-AFAP-120) for 48 h, and immunofluorescence assay was performed using the anti-AFAP-120 antibody. The cells were counterstained with Hoechst33258 (Hoechst). The merged images (Merge) are overlays of the first three panels. Scale bars equal 30 µm.

To detect whether anti-AFAP-120 could recognize the over-expressed human AFAP-120 protein, we transfected plasmids pCMV-Flag-human AFAP-120 and pCMV-Flag-human AFAP-110 into HEK293T cells, respectively. Forty-eight hours after transfection, the cells were lysed, and the extracted proteins were immunoblotted with antibodies against AFAP-120 and Flag tag. Rabbit pre-immune serum was used as a negative control. The results showed that anti-AFAP-120 could react with the Flag-human AFAP-120 protein, but not with the Flag-human AFAP-110 protein, although both Flag-tagged human AFAP-120 and AFAP-110 could be recognized by the anti-Flag antibody (Figure 4A). Neither over-expressed human AFAP-120 nor AFAP-110 could be immunoblotted by the pre-immune serum (Figure 4A).

Next, we detected whether the anti-AFAP-120 antibody could recognize endogenous human AFAP-120 proteins in mammalian cell lines. HEK293T and SH-SY5Y cells were lysed and the extracted proteins were subjected to an immunoblotting assay with anti-AFAP-120 and pre-immune serum, respectively. The results indicated that, in the equivalent whole cell proteins, AFAP-120 proteins were immunoblotted in the SH-SY5Y cells by anti-AFAP-120, but not in the HEK293T cells (Figure 4B). Endogenous human AFAP-120 in the cells could not be immunoblotted by pre-immune serum (Figure 4B).

To further confirm recognition between the anti-AFAP-120 antibody and human AFAP-120 proteins, COS-7 cells were infected with recombinant lentiviruses expressing GFP or GFP-human AFAP-120 for 48 h, and the immunofluorescence assay was performed using the anti-AFAP-120 antibody. The results indicated that molecules labeled by the anti-AFAP-120 antibody had the same location as GFP-AFAP-120 (Figure 4C). However, when lentiviruses GFP-infected cells were labeled by the anti-AFAP-120 antibody, no immunofluorescence signals were detected (Figure 4C).

### 2.5. Reaction of the Anti-AFAP-120 Antibody with Native Human AFAP-120 Protein

**Figure 5:**
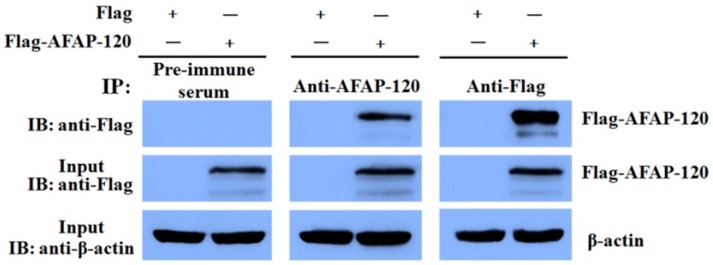
Recognization of anti-AFAP-120 antibody to native human AFAP-120 protein. HEK 293T cells were transfected with plasmids pCMV-Flag-human AFAP-120 (Flag-AFAP-120) or empty vector pCMV-Flag (Flag). Twenty-five micrograms of whole cell protein lysate was used as input to confirm the expression of the Flag-AFAP-120 (with anti-Flag) or β-actin (with anti-β-actin) by immunoblotting (IB). The rest of cell lysates were incubated with anti-AFAP-120, anti-Flag, or pre-immune serum, respectively. The immunoprecipitated (IP) protein complexes were resolved by SDS-PAGE and probed with antibodies against Flag.

An immunoprecipitation experiment was carried out to test the activity of the anti-AFAP-120 polyclonal antibody against the native human AFAP-120 protein. Plasmids pCMV-Flag-human AFAP-120 and empty vector pCMV-Flag were transfected into HEK293T cells. Immunoprecipitation and Western blot analysis were performed on cell lysates from these transfected cells. As shown in Figure 5, Flag-AFAP-120 could be immunoprecipitated by both anti-Flag and anti-AFAP-120. However, it could not be detected in the pre-immune serum-immunoprecipitated proteins.

## 3. Discussion

As an adaptor protein, one of the functions of AFAP-110 is to localize kinases involved in the organization of actin cytoskeleton [3,14,16]. In addition, AFAP-110 can affect the integrity of actin cytoskeleton through crosslinking actin filaments [5,6,7,10,12]. The roles of AFAP-110 in oncogenesis have been examined and found that the loss of AFAP-110 in prostate cancer cells could reduce the rate of proliferation and orthotopic tumor formation in nude mice [19]. For AFAP-120, previous research indicated that it plays a role in organizing dynamic actin structures during neuronal differentiation and suggested that AFAP-120 may help regulate the transition from motile precursor to morphologically differentiated neurons [2]. Those differences imply that AFAP-120 may play different roles from AFAP-110 in the nervous system. In order to further uncover the functions of the AFAP-120 protein, a highly sensitive and specific antibody against AFAP-120 is indispensable. However, up to now, the anti-AFAP-120 antibody is still commercially unavailable, which may hinder the function research for AFAP-120.

The use of KLH and bovine serum albumin conjugated peptides to prepare anti-peptide antibodies has increased dramatically in recent years. Compared with using proteins as antigens, using synthetic peptides as antigens has the advantages of ready availability and the ease of producing the anti-peptide antibody specifically against protein isoforms or site-specific phosphorylated proteins [20]. Therefore, it is feasible that the use of synthetic peptides derived from the NINS fragment to generate an antibody against AFAP-120 protein.

Using experimental methods to characterize epitopes is time-consuming and demands a large amount of resources. The availability of epitope prediction methods can aid experimenters in simplifying this procedure. The ABCpred online software and the BepiPred 1.0 server are two popular B-cell epitopes prediction tools. To obtain high sensitivity, specificity, and positive prediction values, we simultaneously used the ABCpred online serve and the BepiPred 1.0 server to predict B-cell epitopes in the exclusive NINS sequence of the human AFAP-120 protein [21,22], and found a 16aa-peptide sequence (SNHYKYPASAQSVTNT) was the consensus epitope predicted by both tools. This 16aa-peptide was chemically synthesized and used as an immunogen to immunize rabbits. After an antiserum was produced successfully, the specificity and sensitivity of the developed AFAP-120 antibody were detected. The results showed that this antibody could react with the over-expressed and endogenous AFAP-120 protein under a denatured condition, but not with the AFAP-110 protein. Moreover, native AFAP-120 protein was also verified to be recognized by this AFAP-120 antibody with immunoprecipitation.

## 4. Materials and Methods

### 4.1. Plasmid Constructs

The DNA sequence encoding the human AFAP-110 protein was amplified by PCR from the human Schwann cell cDNA library (Beijing, China). The sense primer was 5’-CCGCTCGAGCGGATGGAAGAGTTAATAGTTGAACT-3’, including a *Xho*I site, and the antisense primer was 5’-CCCAAGCTTGGGGTCCCGTTCTTCAATT-3’, including a *Hind* III site. The cDNA sequence encoding the human AFAP-120 protein was synthesized by Sangon Biotech Co., Ltd. (Shanghai, China) and was amplified by PCR using the same primer as described above. Flag-AFAP-110 and Flag-AFAP-120 were constructed by inserting a PCR amplified fragment into the pCMV-Flag vector. The DNA sequence encoding the 84 amino acids of human NINS was amplified by PCR from the plasmid pCMV-Flag-AFAP-120 and was then inserted into the pCMV-Flag vector. The inserted fragment sequences in recombinant plasmids were verified by DNA sequencing (Sangon Biotech Co., Ltd., Shanghai, China).

### 4.2. Sequence Analysis and B-Cell Epitopes Prediction of the AFAP-120 Protein

Firstly, the amino acid sequences of the human AFAP-120 and AFAP-110 proteins were aligned with DNAMAN software (Lynnon Biosoft, San Ramon, CA, USA), and the unique sequences in the AFAP-120 protein were found. The ABCpred online server (http://www.imtech.res.in/raghava/abcpred/) [21] and the BepiPred 1.0 server (http://www.cbs.dtu.dk/services/BepiPred/) [22] were used to predict B-cell epitopes in this unique sequence of the AFAP120 protein, respectively. The ultimate consensus epitope predicted by both tools was synthesized (Sangon, Shanghai, China) and used as an immunogen.

### 4.3. Immunization and Production of the AFAP120-Reactive Rabbit Polyclonal Antibody

One male rabbit (2.5kg) was injected subcutaneously with the immunogen in Freund’s complete adjuvant (FCA) (Sigma, St. Louis, MO, USA) and Freund’s incomplete adjuvant (FIA) (Sigma, St. Louis, MO, USA) in 2-week intervals. The primary immunization consisted of 800 μL immunogen (1 µg/µL, dissolved in PBS) mixed with an equal volume of FCA. For the subsequent immunizations, 400 µL (1 µg/µL, dissolved in PBS) of the immunogen was mixed with an equal volume of FIA. After 4 immunizations, the antiserum was harvested and subjected to affinity purification (ABclonal Biotech, Shanghai, China). Rabbit serum collected before the day of the first immunization was applied as a negative control.

### 4.4. Cell Culture and Transfection

HEK293T, SH-SY5Y, and COS-7 cells were cultured in Dulbecco’s modified Eagle’s medium (Invitrogen, Waltham, MA, USA) supplemented with 10% fetal bovine serum (Invitrogen, Waltham, MA, USA), 2 mM glutamine, and 1% penicillin/streptomycin (Sigma, St. Louis, MO, USA) in a 5% CO_2_ atmosphere at 37 °C. Transfections were performed with Lipofectamine 2000 (Invitrogen, Waltham, MA, USA) following the manufacturer’s protocol.

### 4.5. Immunoprecipitation

Cells were harvested at 48 h post-transfection and lysed respectively in IP Lysis Buffer (Thermo, Waltham, MA, USA) (25 mM Tris·HCl pH 7.4, 150 mM NaCl, 1% NP-40, 1 mM EDTA, 5% glycerol) supplemented with protease and phosphatase inhibitors (Roche, Basel, Switzerland). After the protein concentration of each sample in triplicate was determined using the BCA Protein Assay Kit (Thermo, Waltham, MA, USA), the sample (1 mg) were incubated with 3 µg rabbit anti-Flag polyclonal antibody (MBL, Woburn, MA, USA) or 3 µg rabbit anti-AFAP-120 polyclonal antibody in 1 mL IP Lysis Buffer for 8 h at 4 °C, and the immune complexes were precipitated with 20 µL Protein A/G Plus-agarose (Roche, Basel, Switzerland). The immunoprecipitates were then separated by 12% SDS–polyacrylamide gel electrophoresis.

### 4.6. Immunoblotting

Cells were harvested at 48 h post-transfection and lysed in RIPA lysis buffer (50 mM Tris, pH 7.4, 150 mM NaCl, 1% NP-40, 0.1% SDS) containing a protease inhibitor cocktail. The immunoprecipitates or cells extract proteins were separated by 12% SDS–polyacrylamide gel electrophoresis (SDS-PAGE) and blotted onto a nitrocellulose membrane (Osmonics, Minnetonka, MN, USA) using a semidry blotting apparatus (Bio-Rad, Hercules, CA, USA). The membranes were first blocked with 5% skimmed milk in washing buffer (20 mM Tris, 150 mM NaCl, 0.05% Tween-20, pH 7.6) at room temperature for 1 h and incubated first with primary antibodies at 4 °C for overnight and then with appropriate horseradish peroxidase-labeled secondary antibodies (Santa Cruz Biotechnology, Santa Cruz, CA, USA) at room temperature for 1 h after a few washes. The blots were visualized using an enhanced chemiluminescence immunoblotting detection kit (GE Healthcare, Buckinghamshire, UK).

### 4.7. Immunofluorescence

COS-7 cells were infected with recombinant lentiviruses expressing GFP or GFP-human AFAP-120 (Genechem, Shanghai, China). Forty-eight hours after infection, cells were fixed with 4% paraformaldehyde at room temperature for 30 min and permeabilized in 0.3% Triton X-100. The samples were incubated with the rabbit anti-AFAP-120 polyclonal antibody at 1:2000 dilution at 4 °C for overnight, then stained with a TRITC-conjugated secondary antibody (Abcam, Cambridge, MA, USA) at room temperature for 1 h. The cells were further counterstained with 1 µg/mL Hoechst33258 (Sigma, St. Louis, MO, USA) in PBS and visualized using a confocal laser scanning microscope (Leica, Wetzlar, Germany).

## 5. Conclusions

In summary, to develop an antibody recognizing human AFAP-120, we aligned the amino acid sequences of the human AFAP-120 and AFAP-110 proteins, and a unique NINS fragment in the AFAP-120 protein was found. B-cell epitopes in the NINS sequence was simultaneously predicted by the ABCpred online server and the BepiPred 1.0 server. A 16aa-peptide sequence screened out by both tools was chemically synthesized and used as an immunogen to develop a polyclonal antibody against AFAP-120. The combined results of immunoblotting, immunoprecipitation, and immunofluorescence analyses demonstrated that the AFAP-120 antibody could react with denatured and native human AFAP-120 proteins, but not with the AFAP-110 protein, which may be a useful tool for studying the functions of the AFAP-120 protein.

## Figures and Tables

**Table 1:** Purification analysis of synthetic peptide.

No.	Retention Time	Peak Area	Content (%)
1	8.190	118,254	2.6289
2	8.393	4,283,028	95.2168
3	8.944	96,906	2.1543
Total		4,498,188	100

## Abbreviations

AFAP-120	actin filament-associated protein-120kD
AFAP-110	actin filament-associated protein-110kD
NINS	neuronal insert
PKCα	protein kinase C alpha
Lzip	leucine zipper
MS	mass spectrum
HPLC	high performance liquid chromatography
FCA	Freund’s complete adjuvant
FIA	Freund’s incomplete adjuvant
SDS-PAGE	SDS–polyacrylamide gel electrophoresis
IB	immunoblotting
IP	immunoprecipitation
